# 2G-lactic acid from olive oil supply chain waste: olive leaves upcycling via *Lactobacillus casei* fermentation

**DOI:** 10.1007/s00253-024-13217-z

**Published:** 2024-06-18

**Authors:** Irene Gugel, Filippo Marchetti, Stefania Costa, Ilenia Gugel, Erika Baldini, Silvia Vertuani, Stefano Manfredini

**Affiliations:** 1 Department of Life Sciences and Biotechnology, University of Ferrara, Via L. Borsari 46, 44121 Ferrara, Italy; 2 Department of Chemical, Pharmaceutical and Agricultural Sciences, University of Ferrara, Via L. Borsari 46, 44121 Ferrara, Italy

**Keywords:** Upcycling, Circular economy, Olive leaves, Lactic acid, Biophenols-based products, Fermentation technology

## Abstract

**Abstract:**

The transition towards a sustainable model, particularly the circular economy, emphasizes the importance of redefining waste as a valuable resource, paving the way for innovative upcycling strategies. The olive oil industry, with its significant output of agricultural waste, offers a promising avenue for high-value biomass conversion into useful products through microbial processes. This study focuses on exploring new, high-value applications for olive leaves waste, utilizing a biotechnological approach with *Lactobacillus casei* for the production of second-generation lactic acid. Contrary to initial expectations, the inherent high polyphenol content and low fermentable glucose levels in olive leaves posed challenges for fermentation. Addressing this, an enzymatic hydrolysis step, following a preliminary extraction process, was implemented to increase glucose availability. Subsequent small-scale fermentation tests were conducted with and without nutrient supplements, identifying the medium that yielded the highest lactic acid production for scale-up. The scaled-up batch fermentation process achieved an enhanced conversion rate (83.58%) and specific productivity (0.26 g/L·h). This research confirms the feasibility of repurposing olive waste leaves for the production of lactic acid, contributing to the advancement of a greener economy through the valorization of agricultural waste.

**Key points:**

*• Olive leaves slurry as it did not allow L. casei to ferment.*

*• High concentrations of polyphenols inhibit fermentation of L. casei.*

*• Enzymatic hydrolysis combined to organosolv extraction is the best pretreatment for lactic acid production starting from leaves and olive pruning waste.*

**Graphical Abstract:**

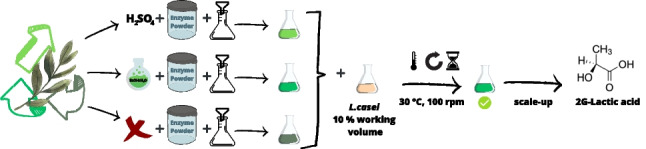

**Supplementary Information:**

The online version contains supplementary material available at 10.1007/s00253-024-13217-z.

## Introduction

The linear economic model, dependent on finite resources and generating excessive waste, is increasingly associated with global crises such as climate change and biodiversity loss (Keijer et al. 2019). In response, the circular economy model advocates for prolonging product life spans, reducing waste, and closing industrial loops to mitigate these issues (Stahel 2016; Kirchherr et al. 2017). Upcycling stands out as a prime example of a circular economy, striving to repurpose objects and materials for “alternative use” to create value-added products (Bridgens et al. 2018). It challenges the notion that waste materials are without value (Wegener 2016), transforming unrecycled organic waste into energy or biochemicals, and considers both post-consumer and production waste valuable resources (Morseletto 2020; Velenturf and Purnell 2021). Plant biomass, abundant in cellulose, hemicellulose, and lignin, is a viable feedstock for sustainable energy and chemical production (Schilling 2019; Velvizhi et al. 2022). Biotechnology is key in reusing waste, facilitating the transition to a circular economy. Recent decades have seen significant promise in fermentation technology for converting agro-industrial biomass into bio-based products, aligning perfectly with the goals of a circular economy and upcycling (Ewing et al. 2022; Singh et al. 2022).

Specific enzymes have been successfully produced from agricultural residues like wheat and rice straw through solid-state fermentation, demonstrating the practical application of circular principles in waste conversion (Kumla et al. 2020).

In the same way, starch, fruit, and vegetable wastes such as orange and banana peel have been used in the biotechnological production of surfactants by exploiting different bacterial species (Domínguez Rivera et al. 2019).

However, there are agro-industrial waste and by-products that have been studied less for their use as a fermentable matrix; olive leaves are one of these under valorized residues derived from both olive tree cultivation and olive oil industry. They are usually removed by burning or grinding and scattering them on fields; due to the large quantities generated each year, it represents an environmental problem (Lama-Muñoz et al. 2020).

The olive oil industry in Europe generates approximately 11.8 million tons of agricultural waste annually (Stempfle et al. 2021). Research by Lama-Muñoz et al. on olive leaf cultivars reveals that from 500,000 tons of leaves, 76,000 tons of cellulose and 45,000 tons of hemicellulose can be extracted each year in the world (Lama-Muñoz et al. 2020).

Since olive leaves have already been described as a lignocellulosic waste, studying its potential as a fermentable matrix could redeem its value. From the best of our knowledge, in the biorefinery scenario, olive tree leaves have so far only been studied as a substrate to produce bioethanol, biogas, and enzymes, but there are no studies on the use of olive tree leaves as a substrate for fine chemicals such as lactic acid (Aydınoğlu and Sargın 2013; Solarte-Toro et al. 2018; Najafi et al. 2021).

Lactic acid is a three-carbon platform chemical (Son et al. 2022) whose market size is expected to grow up to USD 5.80 billion in 2030 (Grand View research report 2024).

However, one of the main challenges in obtaining an economical lactic acid is the choice of substrate. For this reason, the use of low-cost non-food substrates, such as lignocellulosic biomass, has been strongly promoted (Ahmad et al. 2020).

Among the most studied and used lignocellulosic raw materials in biorefinery are corn residues, brewer’s spent grains, sugarcane bagasse, and spent coffee grounds (Yankov 2022).

Recently, other lignocellulosic matrices have been considered. For example, Derabli et al. have demonstrated that *Opuntia ficus indica* fruit peel can be used for lactic acid production reaching a lactic acid yield and productivity of 0.78 g/g and 0.95 g/L‧h, respectively (Derabli et al. 2022).

In the same way, Marques et al. have shown that cashew apple bagasse can be used to produce 25.5 g/L lactic acid with a yield of 0.65 g/g by using *Lactobacillus plantarum* LAB07 (Junior et al. 2024). Furthermore, Abdel-Rahman et al. reported that soft wood and wine shoots could be used as starting substrates for lactic acid production by *Lactobacillus casei* subsp *rhamnosus* (lactic acid yield and titer 0.83 g/g, 23.75 g/L) and *Lactobacillus pentosus* (lactic acid yield and titer 0.76 g/g, 24.00 g/L), respectively (Abdel-Rahman et al. 2011). Olive leaves are not included among the lignocellulosic matrices already investigated for lactic acid production; moreover, they represent a rather abundant second-generation feedstock worldwide.

On the light of the above-mentioned consideration, this study aimed at upcycling olive waste leaves deriving from pruning of unique still preserved cultivar in the Umbria region (Castello di Monte Vibiano, PG, Italy) through a fermentative biotechnological approach. Since they are a complex lignocellulosic material, it was decided to study them as a possible substrate for the growth of the lactic acid bacterium *Lactobacillus casei* with the perspective of producing second-generation lactic acid.

## Materials and methods

### Reagents, raw material, and instruments

d-( +)-Glucose, lactose, l-lactic acid, gallic acid, Folin-Ciocâlteu reagent, and cellulase from *Aspergillus niger* (130 FPU/g) were purchased from Sigma-Aldrich (St. Louis, USA). Dried olive leaves came from the pruning of ancient cultivar of *Olea europaea* L. (Leccino, Moraiolo, and Frantoio) grown on the Castello di Monte Vibiano (PG), Italy. S4V01 was purchased by a local dairy farm. The HPLC system (Jasco, Easton, MD, USA) was equipped with a pump PU-4180, a refractive index detector RI-4030 (Jasco, Oklahoma City, OK, USA) and UV detector UV-4070 (Jasco, Oklahoma City, OK, USA), and a stainless-steel Rezex ROA-Organic Acid H + (8%) column, 300 × 7.8 mm packed with 8-μm particles (Phenomenex, Torrance, CA, USA). UV–VIS spectrophotometer (ONDA UV-30 SCAN Sinergica Soluzioni S.r.l. Milan, Italy). MiniBio Microbial fermenter (Applikon Biotechnology B.V., Delft, Netherlands). D-/L Lactic acid (D-/L-lactate) (Rapid) Assay Kit (Megazyme Ltd., Auchincruive, Scotland, UK) carrying out the assay following the instructions given by the supplier.

### Characterization of raw material

Ash determination was carried out based on the REL Laboratory Analytical Procedure (Van Wychen and Laurens 2016). The total lipid content was determined following AOAC 2003.05. The lignin was measured by using INNVENTIA—Biorefinery Test Methods L 2:2016; cellulose and hemicellulose content in olive leaves was measured by following the Updegraff method (Updegraff 1969) and a one-step acid hydrolysis (Gao et al. 2014) respectively. The nitrogen content was determined by the Kjeldahl method. Sugars from olive leaves were extracted as reported by Romani et al. (1994) and further analyzed through HPLC.

### Bacterial strains and culture conditions

*Lactobacillus casei* DSM 20011 (ATCC-393) was purchased from Leibniz Institute DSMZ-German Collection of Microorganisms and Cell Cultures GmbH Company. The strain was stored in the de Man, Rogosa, and Sharpe (MRS) medium broth (10.0 g enzymatic digest of casein/L, 8.0 g meat extract/L, 4.0 g yeast extract/L, 20.0 g D ( +) glucose/L, 2.0 g dipotassium hydrogen phosphate/L, 1.0 g Tween80/L, 2.0 g diammonium hydrogen citrate/L, 5.0 g sodium acetate/L, 0.2 g magnesium sulfate heptahydrate/L, 0.04 g manganese sulfate monohydrate/L) with glycerol (50% w/v) at − 20 °C. The working cell bank was kept at 4 °C in MRS agar slants and used for seed cultures. For the inoculum preparation, the strain was cultured on Erlenmeyer flasks containing 50 mL of the MRS medium broth at 30 °C, 100 rpm for 24 h.

S4V01 nutrition booster (169.50 ± 3.22 g/L lactose, 0.976 ± 0.19 g/L nitrogen, 6.1 g/L proteins, 0.166 ± 0.088 mg/L Na, 0.858 ± 0.099 mg/L K, 0.040 ± 0.011 mg/L Ca, 0.160 ± 0.022 mg/L Mg) was used in this study and supplied by Ambrosialab srl, Ferrara, Italy.

### Preliminary fermentation tests

The dried olive leaves were mixed with deionized water in a 1:8 ratio and grinded by a laboratory blade homogenizer. The pH of the resulting olive leaves slurry was adjusted to 6.5 with 10 M sodium hydroxide solution. Preliminary small-scale fermentations were performed using 50 mL of olive leaves slurry in 50-mL flasks and inoculated with 5 mL of the *L. casei* inoculum. The same procedure was carried out by adding increasing quantities of S4V01 medium to the olive leaves slurry (Table S1).

S4V01 is a food industry by-product which contains micro- and macronutrients and provides a second carbon source (lactose). Fermentations were conducted at 30 °C, 100 rpm for 6 days (144 h). Substrate consumption and lactic acid production were analyzed by HPLC. For this purpose, samples (1 mL) were collected every 24 h from the start of the fermentation (T0).

### Pretreatments of olive leaves matrix

In order to increase the amount of fermentable glucose in olive leaves matrix, three different saccharification protocols were performed. First, the olive leaves were added with deionized water in a ratio of 1:8 and grinded by a laboratory blade homogenizer. As reported in Table 1, in the first strategy, olive leaves were subjected to an acid hydrolysis step by adding H_2_SO_4_ 3% v/v at 100 °C for 30 min; subsequently an enzymatic hydrolysis was performed by putting different amounts of a commercial cellulase from *Aspergillus niger* corresponding to 1, 5, 10, and 20 FPU/g of dried olive leaves in 80 mL of hydrolyzed olive leaves slurry at pH 5. The flasks containing the matrix supplemented with the enzyme were incubated at 37 °C with gentle shaking for 5 days (120 h). Then, the matrix saccharification protocol was modified, maintaining a single enzymatic hydrolysis step (second strategy) possibly preceded by an “organosolv extraction process” (third strategy). Briefly, an hydroethanolic mixture (80:20) was used as solvent and added to grinded olive leaves in a ratio of 8:1. The extraction was performed at pH 3, 60 °C for 4 h (Yateem et al. 2014). Afterwards, the olive leaves were recovered, dried, and added with deionized water in ratio of 1:8.

**Table 1:** Pretreatments for increasing the amount of glucose

Pretreatment			
1	H_2_SO_4_ 3% v/v	1, 5, 10, 20 FPU/g of dried olive leaves	-
2	-	20 FPU/g of dried olive leaves	-
3	-	20 FPU/g of dried olive leaves	Hydroethanolic mixture (20:80)

Both protocols were carried out by loading 20 FPU/g of dried olive leaves. Sample withdrawal (1 mL), pre- and post-acid hydrolysis, and along the enzymatic saccharification protocols were analyzed by HPLC monitoring glucose release.

The acid hydrolysis yield was calculated as reported by Neureiter et al. (Neureiter et al. 2002):

$$\%\;\text{Acid hydrolysis yield}= \frac{c*V }{M} *100$$

*c* is the concentration of glucose reached after the acid hydrolysis (g/L), *V* is the final volume (L), and *M* is the amount of dried olive leaves used in the experiment (g).

The saccharification yield was calculated as follows (Poornejad et al. 2013):

$$\%\;\text{Saccharification yield}= \frac{\text{Glucose released }(\text{g})}{\text{Initial cellulose }\left(\text{g}\right)\times 1.111} \times 100$$

The dehydration factor (1.111) is employed to consider the water added to the cellulose chains.

### Small-scale fermentation

The olive leaves subjected to all three saccharification protocols were filtered with a Buckner filter thus obtaining three different olive leaves filtrated media (OLFM) which were used for the subsequent small-scale fermentation assays. Each medium was used as it is and diluted with distilled water in ratios of 1:1, 1:2, and 1:5. The pH of the olive leaves media was adjusted to 6.5 with 10 M sodium hydroxide solution. Fermentations were performed using 50 mL of olive leaves medium in 50-mL flasks and inoculated with 5 mL of *L. casei* inoculum. The flasks were then incubated (30 °C, 100 rpm) for 6 days (144 h). In order to investigate the effect of nitrogen supplementation, the same procedure was adopted to carry out small-scale fermentation tests using OLFM non diluted and diluted in ratios of 1:1, 1:2, and 1:5 added with 10% of S4V01. To evaluate glucose consumption and lactic acid production, samples (1 mL) were collected every 24 h from the start of the fermentation (T0) and analyzed by HPLC.

### Total polyphenol content

To evaluate the total polyphenol content of the samples collected after 6 days of incubation, an adapted and optimized Folin-Ciocâlteu assay was used (Singleton and Rossi 1965). Gallic acid was used as standard to obtain a calibration curve (0–500 ppm). Twenty microliters of each sample were added and mixed with Folin-Ciocâlteu reagent diluted in distilled water. After 5 min of incubation at room temperature, 300 µL of a sodium carbonate solution was added, and the whole mixture was incubated in the dark at room temperature. Finally, after 90 min of incubation, the absorbance is measured at 765 nm using a UV–VIS spectrophotometer. Distilled water was used as blank. The results are expressed as microgram gallic acid equivalents (GAE) per milliliter of sample (µg GAE/mL).

### Susceptibility assessment of *Lactobacillus* casei to olive leaf polyphenols

Olive leaf extract, obtained as a by-product of the organosolv extraction process, was used to evaluate the susceptibility of *Lactobacillus casei* to the contained polyphenols. A stock solution of 50 mg/mL was prepared by completely solubilizing the dried extract in 5% DMSO and sterilizing it through 0.22-μm cellulose acetate filters. Dilutions were made from the stock solution in MRS broth to achieve concentrations within the range of 25 to 7.5 mg/mL. Each dilution was analyzed using the Folin-Ciocâlteu assay to determine the total polyphenol content. Bacterial stain was cultured in MRS broth at 30 °C, 100 rpm for 24 h. An aliquot of *Lactobacillus casei* culture was added to sterilized tubes containing diluted olive leaf extract to final bacterial concentration of 5 × 10^5^ CFU/mL. The tubes were then incubated at 30 °C, 100 rpm for 24 h. After the incubation period, bacterial suspensions were analyzed for bacterial growth by measuring the absorbance (OD_600_). The dilutions prepared from stock solution but not inoculated were used as blank. The MRS broth containing 5% DMSO and MRS broth alone were inoculated as previously described and used as negative and positive control, respectively.

### Scale-up batch fermentation

Olive leaves filtrated medium after organosolv extraction and enzymatic hydrolysis was chosen for the scale-up. Batch fermentation was carried out in a 1000-mL fermenter (500-mL working volume) at 100 rpm, 30 °C, and 5% DO_2_. The pH value was automatically maintained at 6.0 by adding 5 M sodium hydroxide solution. During the batch process, which was performed until the complete consumption of the substrates (96 h), samples were collected every 24 h from the start of the fermentation (T0) and further analyzed by HPLC.

### Analytical and statistical analysis

Sugars (glucose, lactose, xylose, and arabinose) and lactic acid were analyzed by a HPLC system. Samples for the HPLC analysis were centrifugated (10,000 × g for 10 min) and filtered with 0.22-μm cellulose acetate filters. A degassed 0.01 M sulfuric acid solution was used as mobile phase in isocratic condition with a flow rate of 0.6 mL/min. The column temperature was 60 °C (Costa et al. 2020).

Total lactic acid produced (LA_tot_) was calculated by subtracting the initial lactic acid deriving from the inoculum to the total LA in the medium at the end of the fermentation period. The specific productivity (q_LA_) was calculated by dividing the total amount of LA formed per hour of fermentation. Substrate uptake represented the amount of substrate utilized during the fermentation period and expressed as a percentage. Yield of LA (YLA) was calculated from the ratio of the grams of lactic acid produced to the grams of glucose and lactose consumed (g/g). The percentage conversion rate of substrates to lactic acid (C_r_) represented the grams of LA per grams of LA expected through optimal conversion of glucose and lactose, taking into account that in microorganisms carrying out homolactic fermentation, such as *L. casei*, 1 mol of hexose is converted to 2 mol lactic acid. All the experiments were carried out in triplicate and results are reported as the mean values ± standard deviation (SD).

## Results

### Characterization of olive leaves

Lignocellulosic substrates, such as olive leaves, are composed of three types of polymers: cellulose, hemicellulose, and lignin (Garcia-Maraver et al. 2013). In the olive leaves used in this study, lignin was the predominant component (46.63 ± 1.30), followed by cellulose and hemicellulose which were present at 11.02 ± 0.33 and 5.59 ± 0.06% w/w, in line with Lama-Muñoz et al. (2020). The soluble sugars found in olive leaves are equal to 0.50 g/100 g of dried olive leaves. The amounts of arabinose and xylose are similar to those reported by Lama-Muñoz et al. while glucose was lower. The composition of olive leaves is reported in Table S2.

### Preliminary fermentation tests

To evaluate the olive leaves fermentability, preliminary lactic acid fermentation tests were carried out (Table 2).

**Table 2:** Lactic acid fermentation in preliminary fermentation tests

Medium composition	Lactic acid production (g/L)
10% olive leaves slurry + 90% S4V01	12.30 ± 0.76
20% olive leaves slurry + 80% SV401	11.20 ± 0.24
30% olive leaves slurry + 70% S4V01	0.77 ± 0.08
50% olive leaves slurry + 50% S4V01	0
70% olive leaves slurry + 30% S4V01	0
90% olive leaves slurry + 10% S4V01	0
100% olive leaves slurry	0

Utilizing olive leaves slurry as the sole component in the culture medium proved ineffective for supporting the fermentation process by *Lactobacillus casei*. This lack of fermentative activity was also observed in media with higher concentrations of olive leaves slurry than S4V01. Conversely, when the slurry concentration was reduced to below 30%, successful lactic acid fermentation occurred. Specifically, a culture medium containing only 10% olive leaves slurry resulted in the production of 12.30 ± 0.76 g/L of lactic acid, while a medium with 20% slurry yielded slightly less, at 11.20 ± 0.24 g/L. Increasing the slurry concentration to 30% led to a significant decrease in lactic acid production, dropping to 0.77 ± 0.08 g/L. However, in the media where fermentation took place, the lactic acid produced mostly resulted from the conversion of lactose from the addition of S4V01. In fact, without any pretreatments, the glucose from the olive leaves was almost completely bound to cellulose and therefore could not be utilised by *L. casei*.

These preliminary findings indicate that olive leaves alone are not a fermentable matrix for *L. casei*, highlighting some sort of correlation between the amount of olive leaves slurry in the media and the production of lactic acid by the microorganism.

### Pretreatments olive leaves matrix

Since olive leaves contain cellulose in appreciable amounts, a combined acid and enzymatic hydrolysis protocol was performed in this study to increase the fermentable glucose in the olive leaves matrix. However, the acid hydrolysis step did not result in a substantial increase in glucose concentration in fact; the concentration of glucose in olive leaves before acid hydrolysis was 1.03 ± 0.18 g/L and after hydrolysis there was a very low increase (up to 3.83 ± 0.33 g/L; 2.24% yield).

**Fig. 1:**
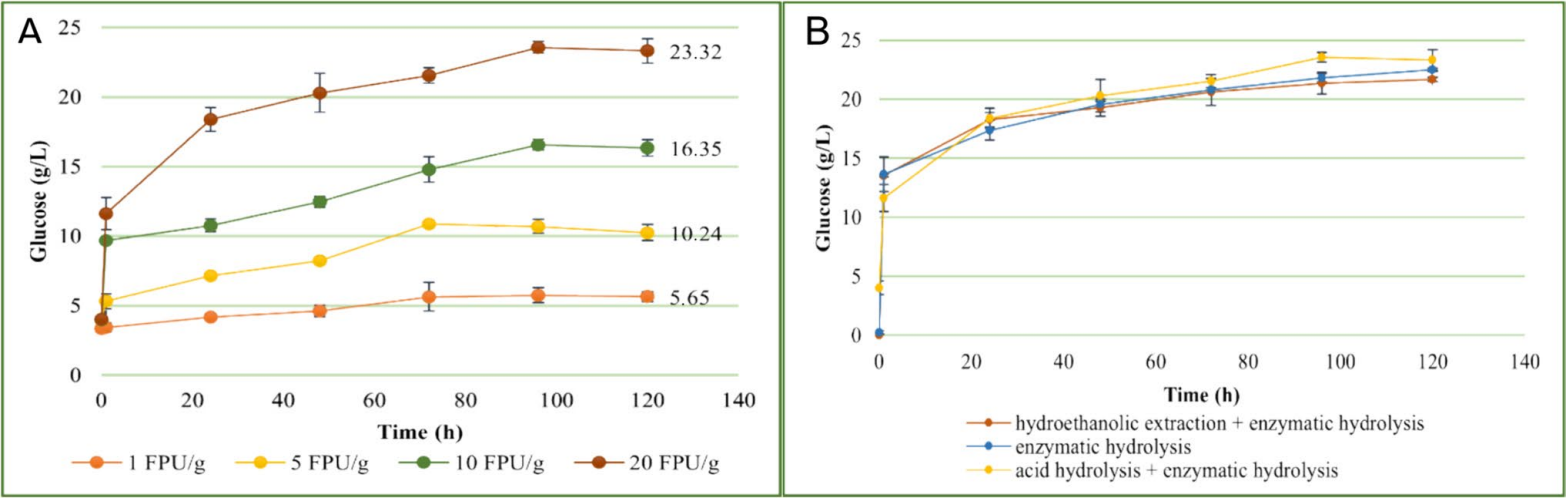
Release pattern of fermentable glucose into the olive leaves matrix after acid hydrolysis at different concentrations (FPU/g of dried olive leaves) of the enzyme cellulase from *Aspergillus niger* (**A**); comparison of the efficacy of the saccharification pretreatments in increasing the release of fermentable glucose into the olive leaves matrix (**B**)

On the hydrolyzed leaves, the enzyme cellulase from *Aspergillus niger* was then added in different quantities to evaluate the release pattern of glucose over time and to identify the most suitable enzyme loading (Fig. 1A). Two other saccharification protocols were investigated by modifying the combined acid and enzymatic hydrolysis protocol. The results obtained (Fig. 1B) were compared to those obtained from the initial protocol consisting of the two hydrolysis steps.

Figure 1A demonstrates the impact of enzyme concentration on glucose release from dried olive leaves. Within the first hour, enzyme concentrations of 10 and 20 FPU/g significantly increased glucose levels to 5.66 g/L and 7.6 g/L, respectively. After 24 h, the glucose concentration further escalated to 18.38 g/L at 20 FP/g, whereas it reached only 10.77 g/L at 10 FPU/g, indicating a pronounced effect of enzyme concentration on glucose release over time. Lower enzyme concentrations, specifically 1 and 5 FPU/g, resulted in considerably lower glucose production at both 1 h and 24 h. The peak of glucose production was observed with 20 FPU/g enzyme loading, achieving around 20 g/L at 96 h, beyond which no further increase was noted.

Conversely, Fig. 1B illustrates that employing three distinct methods of adding *Aspergillus niger* enzyme ultimately yielded comparable glucose concentrations (g/L) after 120 h. The detailed outcomes of utilizing 20 FPU/g enzyme concentration are shown in Table 3.

**Table 3:** Data for the saccharification step with 20 FPU/g of cellulase loading

Pretreatment	Starting glucose (g/L)	Maximum glucose* (g/L)	Yield %
Acid hydrolysis + enzymatic hydrolysis	4.03 ± 0.57	23.32 ± 0.88^a^	53.98%
Enzymatic hydrolysis	0.24 ± 0.14	22.51 ± 0.14^a^	69.31%
Hydroethanolic extraction + enzymatic hydrolysis	0	21.68 ± 0.17^a^	66.28%

*These data take into account a glucose intake of 38.5% resulting from the addition of the enzyme

^a^An enzyme loading of 20 FPU/g dried olive leaves resulted in a glucose supply of 8.80 g/L. The yield (%) was calculated by subtracting the glucose value contributed by the enzyme addition from the maximum glucose value after 120 h of incubation

As Table 3 illustrates, the initial glucose concentrations varied across different strategies. Notably, in the hydroethanolic extraction process, the initial glucose concentration was zero due to a decrease in soluble sugars, which fell from 0.50 ± 0.03 to 0.21 ± 0.02 g/100 g of matrix. Despite this reduction, the low concentrations had no noticeable impact on the final glucose concentration. By the conclusion of the saccharification process, similar glucose titers were observed across all strategies, with the highest yield, 69.31%, achieved through enzymatic hydrolysis alone. This was closely followed by a combination of hydroethanolic extraction and enzymatic hydrolysis, which yielded 66.28%.

In the light of these findings, OLFMs derived from the three saccharification protocols were all tested in small-scale fermentation experiments.

Given the lignocellulosic nature of olive leaves, the presence of pentose sugars such as xylose and arabinose was anticipated. This was confirmed, with concentrations of 4.76 ± 0.2, 2.77 ± 0.09, and 3.18 ± 0.26 g/L for xylose, and 2.31 ± 0.16, 1.21 ± 0.05, and 1.56 ± 0.07 g/L for arabinose, found in OLFMs from acid and enzymatic hydrolysis, enzymatic hydrolysis alone, and organosolv extraction with enzymatic hydrolysis, respectively.

However, consistent with existing literature, *L*. *casei*, the bacterium used in this study, is not naturally capable of fermenting these pentose sugars (Wheater 1955; Chaillou et al. 1999; Chaillou 1999; Acedo-Felix and Pérez-Martínez 2003). Consequently, the potential of these sugars as fermentable substrates was not further explored.

### Small-scale fermentation

The filtrates obtained from olive leaves subjected to the three saccharification protocols (OLFM) were used for small-scale fermentations. Each filtrate was used non diluted and diluted in a ratio of 1:1, 1:2, and 1:5 (Table 4).

**Table 4:** Kinetic parameters calculated from experimental data derived from lactic acid small-scale fermentation tests. For each OLFM without supplementation and OLFM added with 10% S4V01 total lactic acid concentration (LA_tot_), specific productivity (Q_LA_) and substrate uptake (S_up_) are reported

**Without nitrogen supplementation**																								
	No dilution						Dilution 1:1						Dilution 1:2						Dilution 1:5					
	LA_tot_ (g/L)		Q_LA_ (g/L·h)		S_up_% GLU		LA_tot_ (g/L)		Q_LA_ (g/L·h)		S_up_% GLU		LA_tot_ (g/L)		Q_LA_ (g/L·h)		S_up_% GLU		LA_tot_ (g/L)		Q_LA_ (g/L·h)		S_up_% GLU	
**OLFM after acid enzymatic hydrolysis**	0		0		0		0		0		0		5.30		0.04		91.17		4.77		0.03		91.85	
**OLFM after enzymatic hydrolysis**	0		0		0		0		0		0		6.30		0.04		81.32		3.47		0.02		94.56	
**OLFM after organosolv extraction and enzymatic hydrolysis**	0		0		0		5.87		0.04		95.41%		3.44		0.02		100		4.25		0.03		100	
**With nitrogen supplementation**																								
	No dilution						Dilution 1:1						Dilution 1:2						Dilution 1:5					
	LA_tot_ (g/L)	Q_LA_ (g/L·h)		S_up_% GLU		S_up_% LAC	LA_tot_ (g/L)	Q_LA_ (g/L·h)		S_up_% GLU		S_up_% LAC	LA_tot_ (g/L)	Q_LA_ (g/L·h)		S_up_% GLU		S_up_% LAC	LA_tot_ (g/L)	Q_LA_ (g/L·h)		S_up_% GLU		S_up_% LAC
**OLFM after acid enzymatic hydrolysis + 10% S4V01**	0	0		0		0	0	0		0		0	5.67	0.04		80.82		13.99	5.40	0.04		100		22.88
**OLFM after enzymatic hydrolysis + 10% S4V01**	0	0		0		0	0	0		0		0	4.11	0.03		42.29		8.41	4.69	0.03		100		26.42
**OLFM after organosolv extraction and enzymatic hydrolysis + 10% S4V01**	12.44	0.09		54.18		8.93	10.84	0.08		84.80		30.60	8.64	0.06		100		29.44	5.59	0.04		100		26.39

OLFM obtained after a combined protocol of organosolv extraction and enzymatic hydrolysis allowed *Lactobacillus casei* to carry out its own metabolism, producing lactic acid, except the undiluted medium without the addition of supplements. On the other hand, the filtrates obtained by treating the leaves with only enzymatic hydrolysis and enzymatic hydrolysis preceded by acid hydrolysis allowed *L. casei* to produce lactic acid only at dilutions equal to 1:2 and 1:5. Furthermore, the result does not change even by adding to the OLFM a 10% of S4V01, which provides micronutrients and a second carbon source.

The failure of nutritional boosters to make the media conducive for lactic fermentation further supported the hypothesis that olive leaves possess compounds that inhibit the metabolic activity of *Lactobacillus casei*.

However, the effect of dilution on the non-fermentable filtrates allowed lactic fermentation to take place. In all protocols performed, OLMF medium after organosolv extraction and enzymatic hydrolysis proved to be the best performing; in the absence of dilution, it is able to ferment with the addition of the supplement S4V01. In fact, since it is not diluted, it has the highest concentration of starting substrate (glucose and lactose) and at the same time allows the microorganism to carry out its metabolism adequately. Furthermore, this medium allowed the maximum amount of lactic acid produced (12.44 g/L) and the highest volumetric productivity value (0.09 g/L·h), while for the other strategies, the tendency is for better fermentation efficiency as the dilution factor increases.

**Fig. 2:**
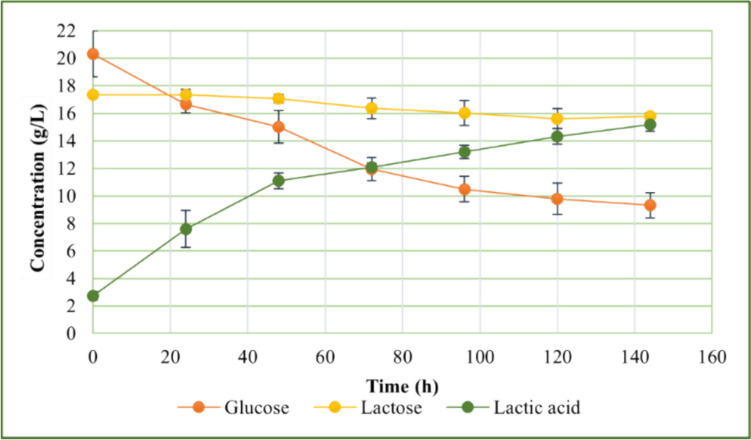
Fermentation trend of OLFM after organosolv extraction and enzymatic hydrolysis with S4V01 supplementation

In Fig. 2, the trend of the small-scale fermentation with OLMF medium after organosolv extraction and enzymatic hydrolysis added with 10% S4V01 is reported.

At the conclusion of the incubation period (144 h), the concentration of lactic acid reached 15.18 g/L; after subtracting the amount of lactic acid produced by the inoculum, the net lactic acid produced in the OLMF medium after organosolv extraction and enzymatic hydrolysis supplemented with S4V01 was 12.44 g/L. Analysis of sugar consumption patterns revealed that *Lactobacillus casei* utilized glucose faster than lactose, with uptake rates of 54.18% and 8.93%, respectively. Neither sugar was completely consumed, likely due to the culture medium becoming excessively acidic (average pH of 3.53 ± 0.08) before the incubation period ended. This acidity, a consequence of lactic acid bacteria fermentation, can inhibit bacterial growth and even lead to cell death, as noted by Zhang et al. (2012).

### Total polyphenol content

**Fig. 3:**
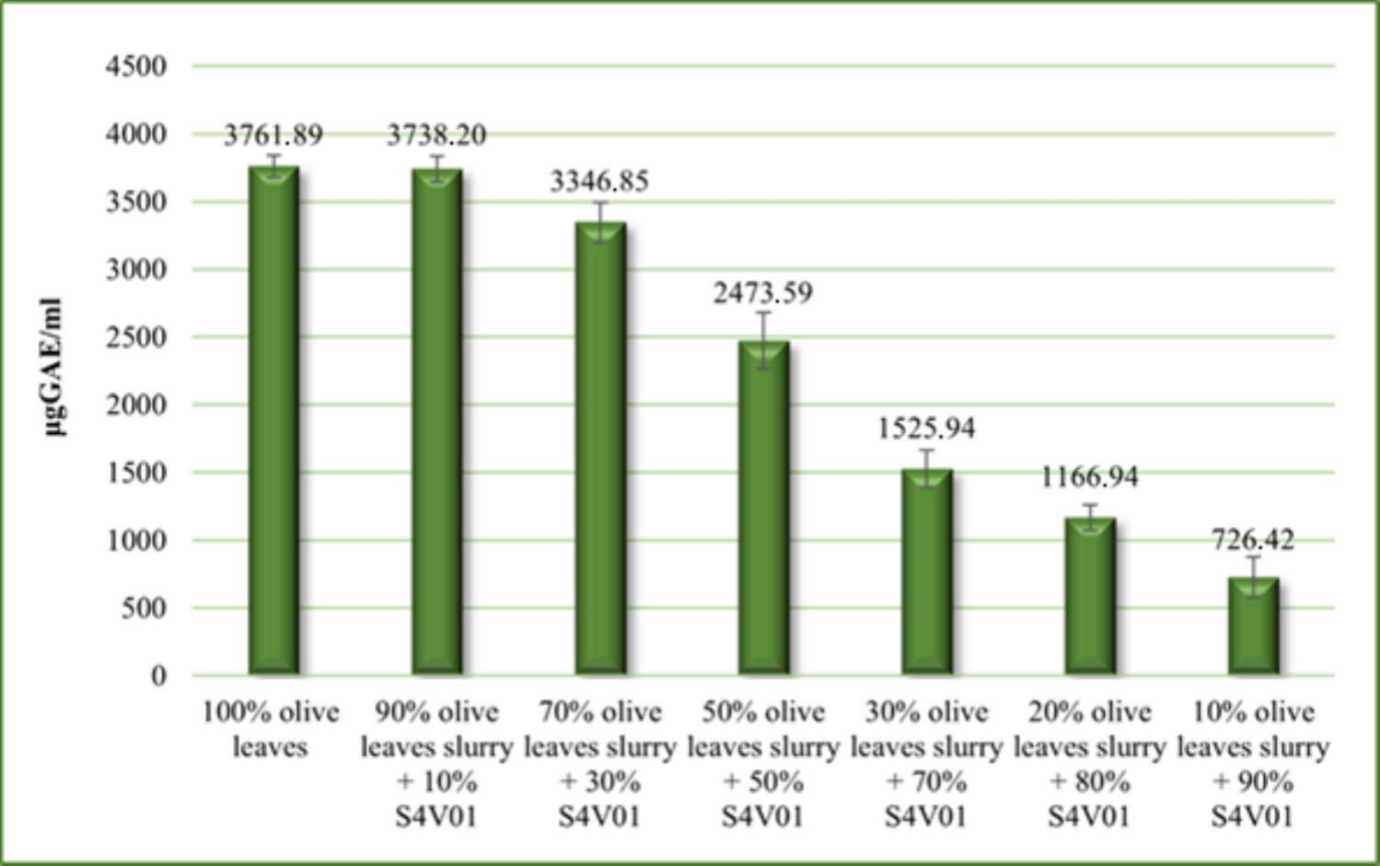
Total polyphenol content in olive leaves slurry media after an incubation time of 144 h

Samples collected from each fermentation test were analyzed for their total polyphenol content to investigate the possible inhibitory effect of olive leaves polyphenols on *L. casei* (Fig. 3).

**Fig. 4:**
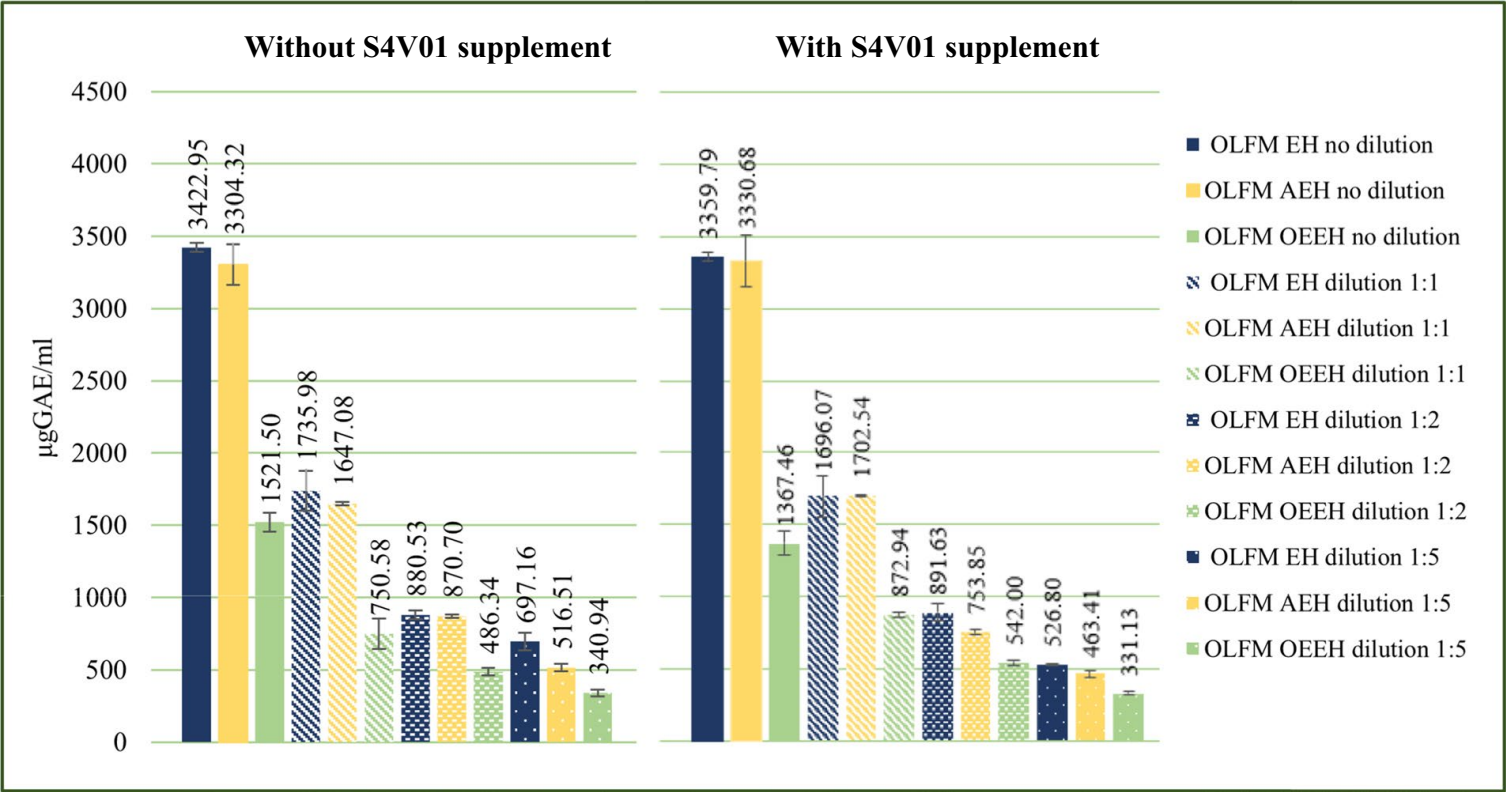
Total polyphenol content in olive leaves filtrate media (OLFM) without (**A**) and added with S4V01 supplementation (**B**) after an incubation time of 144 h: OLFM EH (olive leaves filtrate medium obtained after the enzymatic hydrolysis), OLFM AEH (olive leaves filtrate medium obtained after acid and enzymatic hydrolysis), and OLFM OEEH (olive leaves filtrate medium obtained after the enzymatic hydrolysis preceded by an organosolv extraction)

Figure 3 illustrates that in preliminary fermentation tests, media with higher amounts of olive leaves slurry exhibited higher polyphenol concentrations. Conversely, as the amount of S4V01 in the media was augmented, a decrease in total polyphenol content was observed. However, this decrease was not linearly proportional to the amount of S4V01 added. This anomaly is likely due to the presence of the solid olive leaves in the media, which, when incubated at 30 °C for 6 days in an aqueous medium like S4V01, enhances the leaching of polyphenols from the solid matrix into the liquid phase. This mechanism accounts for the observed non-proportional reduction in polyphenol levels with decreasing olive leaf slurry content in the preliminary tests. Notably, this trend was absent in olive leaves filtrate media (OLFM), where the solid matrix had been removed prior to testing. Figure 4 presents data on the total polyphenol content in OLFM, both with and without S4V01 supplementation, further supporting this hypothesis.

The results demonstrated a clear correlation between the total polyphenol content in the fermentation medium and its dilution level, as indicated by the coefficients of determination (*r*^2^) listed in Table S3. Specifically, media subjected to preventive organosolv extraction, which effectively removes a significant portion of polyphenolic molecules, exhibited lower polyphenol contents. Conversely, media not treated with hydroethanolic extraction contained considerably higher polyphenol levels. It is also important to highlight that the Folin-Ciocâlteu assay was applied to S4V01 alone, revealing a minimal presence of reducing substances (65.289 ± 2.57 µg GAE/mL) capable of reacting with the assay reagent in its undiluted form. However, when diluted to the same concentration used in the small-scale fermentation tests (10% S4V01), no interfering substances were detected. This finding confirms that S4V01 did not interfere with the determination of total polyphenol content in the OLFM fermentation samples.

### Susceptibility assessment of *Lactobacillus* casei to olive leaf polyphenols

To further investigate the susceptibility of *Lactobacillus casei* to olive leaf polyphenols, its growth in the presence of increasing amounts of polyphenols over a 24-h period was monitored.

From Table 5, it is evident that high concentration of olive leaf polyphenols negatively influenced the growth of *L. casei* compared to the control. The highest concentration of polyphenols tested (1799.60 μg GAE/mL) resulted in a significant inhibition of *L. casei* growth, reducing it by 86.21% compared to the positive control. Reducing the polyphenol concentration showed a corresponding decrease in the inhibitory effect. For instance, halving the highest concentration to 920.05 μg GAE/mL decreased growth inhibition to 18.97%. Further reduction in polyphenol content led to even lower inhibition rates: a concentration of 713.30 μg GAE/mL (equivalent to 10 mg/mL of the extract) resulted in 53.45% growth inhibition, while at a concentration of 530.27 μg GAE/mL (7.5 mg/mL of the extract), the inhibition was further reduced to 41.38% relative to the positive control.

**Table 5:** Antimicrobial effect of olive leaf polyphenols on the growth of *Lactobacillus casei*

Olive leaf extract	Total polyphenol content (μg GAE/mL)	OD_600*_
25 mg/mL	1799.60 ± 60.28	0.08 ± 0.03
20 mg/mL	1353.05 ± 35.98	0.13 ± 0.02
15 mg/mL	1066.64 ± 11.08	0.13 ± 0.01
12.5 mg/mL	920.05 ± 19.08	0.19 ± 0.03
10 mg/mL	713.30 ± 53.08	0.27 ± 0.02
7.5 mg/mL	530.27 ± 19.19	0.34 ± 0.07
Ctrl positive	0	0.58 ± 0.02
Ctrl negative	0	0.59 ± 0.06

*Data derived from spectrophotometer readings at a wavelength of 600 nm and expressed as absorbance (OD_600_)

### Batch fermentation

**Fig. 5:**
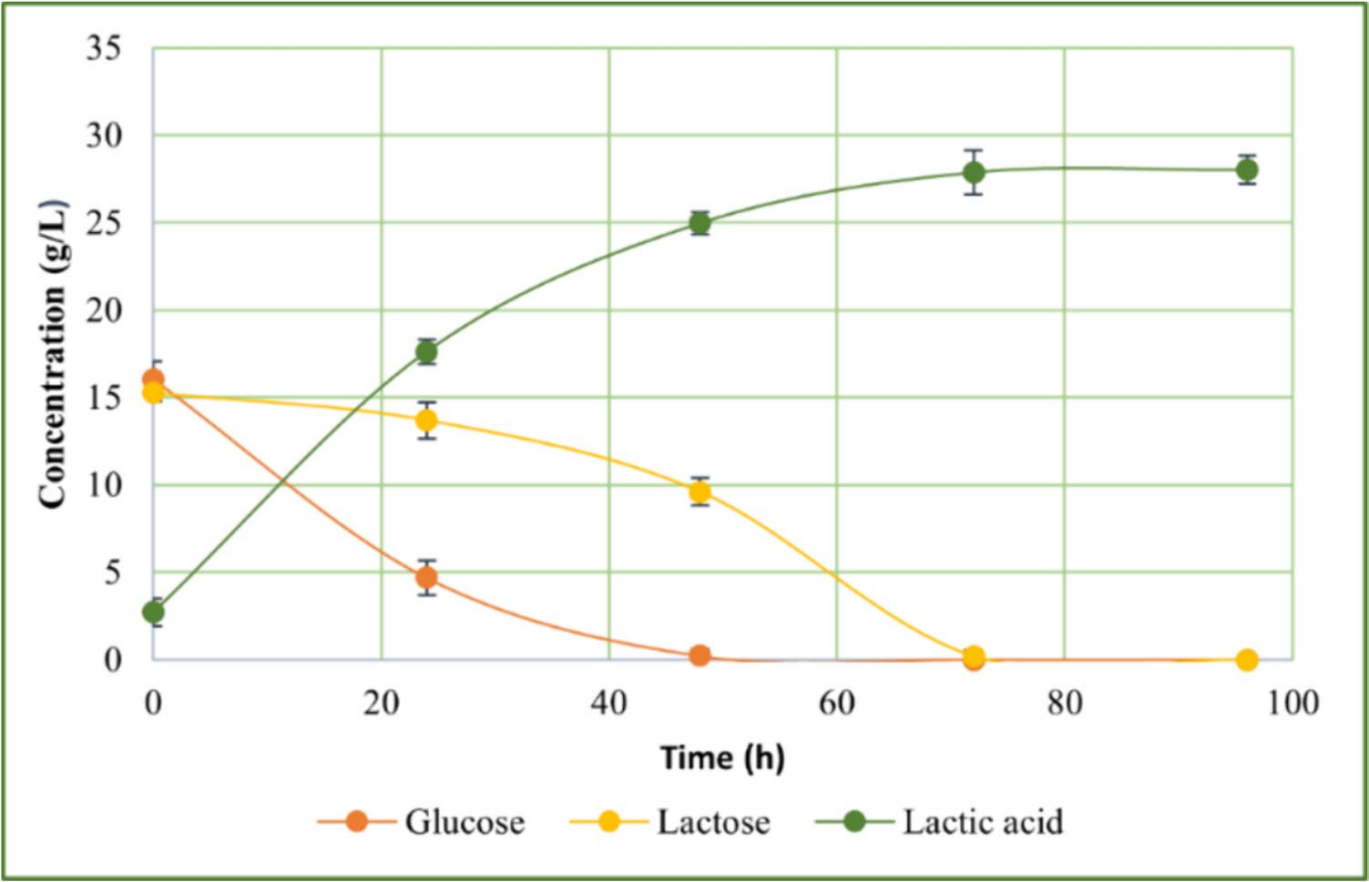
Batch fermentation of *Lactobacillus casei* using OLFM OEEH + 10% S4V01 as starting medium for lactic acid production

Figure 5 shows the trend of batch fermentation using OLFM as starting medium obtained by subjecting olive waste leaves to organosolv extraction and enzymatic hydrolysis supplemented with 10% of S4V01.

Unlike in small-scale fermentation tests, both glucose and lactose were completely consumed in the batch fermentation process, although their consumption rates were different. *L. casei* metabolized glucose more rapidly than lactose, a discrepancy attributed to the bacterium’s need to reorganize its enzyme pool to hydrolyze the disaccharide lactose and metabolize the monomers glucose and galactose (Alvarez et al. 2010; Dedenaro et al. 2016).

Furthermore, the batch fermentation was completed in a shorter timeframe of only 96 h, compared to the duration of small-scale fermentations. Examining the kinetic parameters (Table S4), the batch fermentation process resulted in a higher lactic acid titer than the small-scale tests, with an increase in productivity from 0.09 (in small-scale fermentation) to 0.26 (in batch fermentation). The conversion rate was 83.58%, with a yield (Y_LA_) of 0.63 g/g, culminating in a lactic acid production of 25.29 g/L. Notably, the lactic acid generated was predominantly in the L-form, with an enantiomeric purity of 92.54 ± 2.10%.

## Discussion

As an alternative to the current linear economy based on the “extract, produce, use and throw away” scheme, the circular economy is a model of production and consumption that aims to extend the life cycle of waste materials as long as possible, while minimizing the generation of waste (European Commission 2020), a representative and virtuous model of the circular economy, early introduced by the chemist Michael Braungart around 1995 and lately consolidated with the architect William McDonough that culminated with their famous book (McDonough and Braungart 2010) and the visionary foundation of the Cradle to Cradle Institute and certification system. Crucial was also the idea of is upcycling (McDonough and Braungart 2013) through which waste materials can be used to obtain value-added products (Petit et al. 2022).

The olive oil industry in European countries generates approximately 11.8 million tons of agricultural residues annually (Stempfle et al. 2021), posing environmental challenges if not managed properly (Espeso et al. 2021). Despite this, these residues, especially olive leaves, are recognized for their health benefits (Vogel et al. 2015; Özcan and Matthäus 2017) and represent a significant biomass resource. Recent research has explored the valorization of olive leaves for biofuels, bioethanol, animal feed, and agricultural uses (Stempfle et al. 2021). However, their potential for producing higher-value products, such as pharmaceuticals or fine chemicals, remains largely untapped. Currently, the extraction of bioactive compounds, mainly polyphenols, is the primary high-value application of olive biomass (Berbel and Posadillo 2018). Given the wide industrial applications of lactic acid in the food, pharmaceutical, and cosmetic industries (Vijayakumar et al. 2008), this study proposes exploring olive leaves as a novel substrate for producing lactic acid as a method of upcycling this abundant agricultural residue.

For lactic acid production, numerous studies have identified bacteria and fungi with high yields and productivity. For example, strains of *Lactobacillus rhamnosus* and *Lactobacillus delbrueckii* have shown promising results. Additionally, genetically engineered yeast strains like those from the *Saccharomyces cerevisiae* species have been modified to improve lactic acid production (Balasubramanian et al. 2024). However, taking advantage from previous results concerning *Lactobacillus casei* fermentation on non-lignocellulosic food waste (Dedenaro et al. 2016), we decided to test it even on second-generation feedstocks.

In the preliminary fermentation tests, the medium composed only of olive leaves slurry did not allow the production of lactic acid. Therefore, small-scale fermentation tests were performed using media composed of different percentages of olive leaves slurry supplemented with S4V01, which could be considered as a nitrogenous source and at the same time provides a second carbon source (lactose) usable by the bacterium *L. casei*. We observed that the lactic acid fermentation was successful in this case, but only for media containing percentages of olive leaves slurry lower than 30%.

Indeed, an accurate literature analysis revealed that compounds with antioxidant activity deriving from vegetable matrices can exert a bactericidal or bacteriostatic effect against the bacterium *Lactobacillus casei* (Duda-Chodak et al. 2008). For this reason, the Folin-Ciocâlteu assay was performed. A correlation between the concentration of polyphenolic compounds in the medium and the lack of lactic acid production was observed.

From the results obtained in these preliminary tests, two main obstacles emerged: a low amount of glucose intrinsically present in the olive leaves matrix and the presence of substances inhibiting the bacterial metabolism, probably the polyphenols. Therefore, pretreatments aimed at increasing the quantity of fermentable glucose and at reducing the concentration of polyphenols in the plant matrix were investigated.

The release of high amounts of sugar monomers can be achieved by breaking the glycosidic bonds of the lignocellulosic biomass by acid hydrolysis (Loow et al. 2016; Świątek et al. 2020).

Thus, the leaves of olive trees were subjected to a pretreatment of acid hydrolysis followed by a pretreatment of enzymatic hydrolysis.

In fact, lignocellulosic matrices can be hydrolytically decomposed into monomers both chemically, using strong acids, and by exploiting cellulolytic enzymes (Maitan-Alfenas et al. 2015). According to Narayanaswamy et al., enzymatic hydrolysis is more effective if preceded by a pretreatment of the matrix, which breaks the crystal structure of lignocelluloses exposing the cellulose and hemicellulose molecules to a more efficient enzymatic saccharification (Narayanaswamy et al. 2013).

In the study, cellulase from *Aspergillus niger* was applied to olive leaves slurry at varying levels (1, 5, 10, and 20 FPU/g of dried olive leaves) following an acid hydrolysis step. The optimal glucose yield was observed with the highest enzyme concentration of 20 FPU/g, which increased the fermentable glucose from 4.03 ± 0.57 g/L to 23.32 ± 0.88 g/L over 120 h. This finding aligns with research by Láinez et al. (2018) on *Agave salmiana* leaves, suggesting that greater enzyme amounts enhance glucose release. Nonetheless, due to the high cost of the enzyme and the imperative to minimize expenses for industrial application (da Silva et al. 2020), this study did not explore beyond this enzyme concentration, despite the potential for higher glucose yields. Remarkably, the achieved glucose concentration was comparable to that in the de Man-Rogosa-Sharpe (MRS) synthetic medium traditionally used for lactobacilli cultivation.

Next, it was chosen to evaluate whether the enzymatic saccharification step alone was sufficient to increase the concentration of glucose. In fact, the only acid hydrolysis step does not contribute significantly to the increase of fermentable glucose in the matrix. Also, the use of sulfuric acid as a catalyst has long been associated with degradation by-products such as furfural, hydroxymethylfurfural (HMF), and acetic acid, which can inhibit the fermentation process (Loow et al. 2016).

The enzymatic hydrolysis was also carried out preceded by an extraction process. According to Diaz et al., the organosolv treatment of the olive pruning biomass allows to improve the subsequent enzymatic hydrolysis step (Díaz et al. 2011). For this reason, we performed the enzymatic hydrolysis preceded by an idro-ethanolic extraction in order to make the matrix more accessible to cellulase enzyme and at the same time to promote the removal of molecules probably responsible for the inhibition of the *L. casei* metabolism.

Unlike what we expected, comparing the results obtained from the pretreatments of the olive leaves matrix, it was possible to notice how all three pretreatments lead to very similar results in terms of increase in glucose concentration (range between 21.68 and 23.32 g/L) and in saccharification yields (53.98–69.31%). Concerning the percentage yield, lower results were reported by Alrumman Sulaiman who obtained a maximum saccharification degree of 19.57% on acid-steam-treated date palm leaves. An increase in saccharification percentage yield (41.40%) was obtained instead by pretreating the date palm leaves with alkaline hydrolysis (Alrumman 2016).

Therefore, each of the filtrates (OLFM) obtained from the three pretreatment protocols was tested as a potential medium for lactic acid production. It was decided to use both undiluted and diluted filtrates in order to reduce the concentration of inhibitory substances. The same tests were carried out by adding to the medium 10% of S4V01. It is generally recognized that some micronutrients, such as Mn^2+^, Mg^2+^, Ca^2+^, Fe^2+^, K^+^, and Na^+^, are required by lactic acid bacteria as they act as essential factors or stimulators for nutrient transport and enzymatic activity (Saeed and Salam 2013). In particular, it has been observed that potassium is necessary for the growth of *Lactobacillus casei* as well as calcium, which acts as a growth-stimulating agent (MacLeod and Snell 1947; Eades and Womack 1953). Most of the above-mentioned minerals are present within the S4V01 nutrient booster. Furthermore, the presence of organic nitrogen and proteins makes S4V01 a source of nitrogen.

The undiluted and 1:1 diluted medium, obtained after subjecting the leaves to the combined acid and enzymatic hydrolysis protocol and to the enzymatic hydrolysis step alone, did not allow *L. casei* to ferment. From the Folin-Ciocâlteu assay data, these media appear to contain the highest concentrations of polyphenols.

On the other hand, when the concentration of total polyphenols decreases in the medium due to dilution, *L. casei* converts the sugar substrate into lactic acid. Also, the non-diluted medium obtained from previously extracted olive leaves (OLFM OEEH) without supplement did not allow *L. casei* to ferment. However, the result changes if we add a booster (S4V01) to the latter medium which, in addition to provide further substrate useful to the bacterium; it helps to dilute the polyphenol content.

Presenting a lower content of total polyphenols, it led to the production of 12.44 g/L and in this medium the bacterium can carry out its metabolism with a productivity of 0.09 g/L·h.

Pereira et al. (2007) established that olive leaves polyphenols inhibit the growth of various bacterial species, yet the specific effects on *Lactobacillus casei* remain less understood. Our susceptibility test confirmed a close correlation between polyphenol concentration and the inhibition of *L. casei* growth, demonstrating increased growth inhibition with higher polyphenol levels. Sudjana et al. (2009) also observed that a commercial olive leaf extract, at concentrations of 12.5 to 25% v/v, inhibits *L. casei*. This suggests that *L. casei* is particularly sensitive to polyphenols, with oleuropein and hydroxytyrosol identified by Peng et al. (2017) as key inhibitory compounds acting in a dose-dependent manner. Despite these insights, research focusing on the effect of olive leaves polyphenols on *L. casei* is scarce, contrasting with the broader body of work available on their impact on other microbial strains (Ghomari et al. 2019; Martín-García et al. 2022; Šimat et al. 2022).

Since lactic acid is a primary metabolite partially associated with bacterial growth, it is possible to deduce that in media (OLFM) containing a higher concentration of total polyphenols, the microorganism replicated less and consequently failed to carry out its primary metabolism.

Due to the inhibitory effect that these molecules can exert on sugar fermentation (Stempfle et al. 2021), an extraction process in the matrix pretreatments helps to bypass this problem.

Finally, we scaled up the fermentation process using the OLFM OEEH + 10% S4V01 which appeared to be the most promising since it contained the largest amount of initial substrate, and which allowed the greatest production of lactic acid in small-scale tests. In 96 h, complete depletion of fermentable sugars occurred, with total lactic acid production of 25 g/L: double compared to flask tests. The conversion rate C_r_ was 83.58% and the specific productivity increased up to 0.26 g/L·h; furthermore, the optical purity of l-lactic acid produced was 92.52%. Although the results reported in this study are lower, in terms of both productivity and lactic acid produced, than what has been reported in the literature using other lignocellulosic substrates such as cassava and sugarcane bagasse, vine trimming waste, and rice straw (John et al. 2006; Rivas et al. 2007; Wischral et al. 2019; Sivagurunathan et al. 2022), it is important to emphasise that this is the first study to evaluate olive leaves as a possible substrate for second-generation lactic acid production. Therefore, further studies will be necessary to improve the fermentability of the starting substrate, to identify the most suitable microorganism and possibly to experiment with other fermentation strategies.

In this research, we wanted to explore an alternative way to recover the olive oil supply chain biomass, specifically by upcycling olive leaves into a valuable resource for biotechnological fermentation to produce lactic acid. While initially aiming to derive lactic acid biophenols-enriched extracts, we faced unexpected challenges due to the inhibitory effects of biophenols on *L. casei* growth. Our approach involved performing pretreatments to enhance fermentable sugar availability, notably increasing the initial glucose concentration to approximately 20 g/L. To mitigate the growth inhibition caused by polyphenolic compounds, a hydroethanolic extraction step was incorporated prior to enzymatic hydrolysis, facilitating a successful scale-up process that improved specific productivity and lactic acid yield. The findings from our lab-scale experiments underscore the feasibility of utilizing olive leaves waste as a starting material for lactic acid production with *L. casei*.

These results could be considered of great interest: olive leaves waste could be upcycled through the biotechnological approach. This study showed for the first time that this waste can be considered as a resource for obtaining fine chemicals and ingredients for the food and cosmetic industries through fermentation, although we are aware that further studies are needed to improve substrate fermentability and increase lactic acid production.

## Supplementary Information

Below is the link to the electronic supplementary material.

Supplementary file1 (PDF 214 KB)

## Data Availability

The data that support the findings of this study are in this published article and its supplementary information file and available from the corresponding author upon reasonable request.

## Code availability

Not applicable for that section.
