# Supplementary material for: 2G-lactic acid from olive oil supply chain waste: olive leaves upcycling via Lactobacillus casei fermentation
Source: Appl Microbiol Biotechnol. 2024 Jun 18;108:379. doi: 10.1007/s00253-024-13217-z (PMC11189319; doi:10.1007/s00253-024-13217-z)
Supplement: Supplementary file 1 [file 253_2024_13217_MOESM1_ESM.pdf]

Supplementary material

Applied Microbiology and Biotechnology

2G-Lactic Acid from Olive Oil Supply Chain Waste: Olive Leaves upcycling via *Lactobacillus casei* Fermentation

Irene Gugel[1,2], Filippo Marchetti[1,2], Stefania Costa[1,2]*, Ilenia Gugel[1], Erika Baldini[1], Silvia Vertuani[1] and Stefano Manfredini[1]

1 Department of Life Sciences and Biotechnology, University of Ferrara Via L. Borsari, 46 Ferrara 44121, Italy;
2 Department of Chemical, Pharmaceutical and Agricultural Sciences, University of Ferrara Via L. Borsari, 46 Ferrara 44121, Italy;

Correspondence: Stefania Costa, cstsfn1@unife.it. Phone: + 39 0532455708

Table S1:

Media composition used for preliminary fermentation tests

	Medium composition
1	10% olive leaves slurry + 90% S4V01
2	20% olive leaves slurry + 80% SV401
3	30% olive leaves slurry + 70% S4V01
4	50% olive leaves slurry + 50% S4V01
5	70% olive leaves slurry + 30% S4V01
6	90% olive leaves slurry + 10% S4V01

Table S2:

Composition of olive leaves

	g/100g dry basis
**Soluble sugars**	0.50 ± 0.03
Glucose	0.13 ±0.01
Arabinose	0.11 ± 0.01
Xylose	0.25 ± 0.01
**Total nitrogen**	1.59 ± 0.07
**Lipids**	14.38 ± 0.37
**Ash**	6.64 ± 0.08
**Lignin**	46.63 ± 1.30
**Cellulose**	11.02 ± 0.33
**Hemicellulose**	5.59 ± 0.06

Table S3:

Coefficient of determination (r^2)

	r^2
OLFM EH	0.9887
OLFM AEH	0.9959
OLFM OEEH	0.9979
OLFM EH + S4V01	0.9953
OLFM AEH + S4V01	0.9864
OLFM OEEH + S4V01	0.9745

Table S4:

Kinetic parameters calculated from experimental data derived from batch fermentation test using OLFM OEEH supplemented with 10% of S4V01 as starting medium. Total lactic acid concentration (LA_{tot}), conversion rate (C_r), lactic acid yield (Y_{LA}), specific productivity (q_{LA}) and substrate uptake (S_{up}) are reported.

	LA_{tot} (g/L)	C_r (%)	Y_{LA} (g/g)	q_{LA} (g/L·h)	S_{up} GLU (%)	S_{up} LAC (%)
OLFM OEEH + 10% S4V01	25.29	83.58	0.63	0.26	100	100
